# The association between serum adiponectin and 3-month outcome after ischemic stroke

**DOI:** 10.1186/s12933-019-0908-z

**Published:** 2019-08-14

**Authors:** Zengliang Wang, Bo Li, Yongxin Wang, Aisha Maimaitili, Hu Qin, Geng Dangmurenjiafu, Shuo Wang

**Affiliations:** 1 Department of Neurosurgery, Beijing Tiantan Hospital, Capital Medical University, Fanyang Road, Fengtai District, Beijing, 100070 People’s Republic of China; 2 Department of Neurosurgery, First Affiliated Hospital of Xinjiang Medical University, Ürümqi, China

**Keywords:** Ischemic stroke, Adipokine, Adiponectin, Functional outcome

## Abstract

**Background:**

Although adiponectin is a major adipocytokine that affects the pathogenesis of various cardiovascular diseases, its clinical significance in stroke remains controversial. The purpose of this study was to assess the impact of serum adiponectin levels on functional prognosis in patients with ischemic stroke.

**Methods:**

This was a prospective, observational cohort study. Consecutive first-ever ischemic stroke patients without any pre-morbid handicap admitted to our hospital were identified from December 2017 to December 2018. Serum concentration of adiponectin was routinely measured within the first 24 h after admission by a commercially available sandwich ELISA. Associations between adiponectin and either clinical severity at admission, poor outcomes or mortality at 3-month after admission were analyzed using logistic regression to obtain odds ratios (OR) and 95% confidence intervals (CI).

**Results:**

The serum level of adiponectin was obtained in 227 patients with a median value of 7.0 μg/ml, which was significantly higher (*P *< 0.001) than in those heathy control. Adiponectin levels were associated with moderate-to-high stroke, and risk increased by 12% (OR = 1.12; 95% CI 1.03–1.25; *P *= 0.002). Patients with a poor outcome and nonsurvivors had significantly increased adiponectin levels on admission (P < 0.001, all). In multivariate logistic regression analysis, adiponectin was an independent predictor of functional outcome and mortality, and risk increased by 24% (OR = 1.24, 95% CI 1.13–1.37; *P *< 0.001) and 31% (1.31 [1.18–1.46], *P *< 0.001), respectively. Kaplan–Meier analysis suggested that the patients with high serum adiponectin levels had a higher risk of death than those patients with low levels (log-rank test *P *< 0.001).

**Conclusions:**

Our results show that high adiponectin is associated with stroke severity and support the hypothesis that adiponectin can be serve as a biomarker of poor outcome after stroke, independent of baseline variables.

*Trial registration* ChiCTR-OPC-17013501. Retrospectively Registered 21 September 2017

## Background

Adipose tissue secretes various pro- and anti-inflammatory adipokines to modulate inflammation and insulin resistance [1]. Furthermore, in overweight pre-diabetics patients the insulin resistance might trigger the pro-inflammatory status by over activity of abdominal adipose tissue [2], and this might cause not only an over expression of inflammatory cytokines [2], but also of other molecules and proteins involved in apoptotic processes as sirtuins [3]. In fact, these molecules involve not only metabolic pathways, but also the cardiac performance, by a distance cross talking effect. Previous preclinical and clinical studies investigated the principle sex hormones, as well as select adipose-derived hormones (adiponectin, leptin, and ghrelin), as risk factors or potential treatments for ischemic stroke [4].

Adiponectin first identified in 1995, constitutes a 30-kD glycoprotein made up of 244 amino residues [5]. Adiponectin is an adipokine that is specifically and abundantly expressed in adipose tissue and directly sensitizes the body to insulin [6]. Human adiponectin is present in three isoforms-high, middle, and low molecular weight adiponectin. Among these, high-molecular-weight adiponectin is considered to be the active form [7]. Adiponectin represents a multifaceted biomarker that may beneficially affect atherosclerosis, inflammation and insulin resistance pathways [8]. In humans, it has been indicated that low adiponectin concentrations associated with metabolic syndrome [9], atherosclerosis [10], hypertension [11] and cardiovascular disease [12]. Adiponectin has protective actions in the initiation and progression of atherosclerosis through anti-inflammatory and anti-atherogenic effects [13]. Ilhan et al. [14] showed that decreased adiponectin levels may be a sign of cerebrovascular disease and as part of the response occurring in stroke patients, while another study demonstrated that decreased adiponectin level at admission might be associated with depression in patients after acute ischemic stroke [15]. In addition, a previous study showed that adiponectin levels were independently associated to restenosis (odds ratios [OR] 0.206; 95% CI 0.053–0.796; P < 0.001), novo ischemic heart disease (OR 0.206; 95% CI 0.053–0.796; P ≤ 0.001) and overall new percutaneous coronary interventions (OR 0.206; 95% CI 0.053–0.796; P < 0.001) in normal glucose tolerance patients undergoing percutaneous coronary intervention [16]. The link between adipokines plasma levels, insulin resistance and ischemic heart disease had been proposed [16].

Furthermore, higher circulating adiponectin at baseline had been suggested as an independent risk factor for the development of new-onset atrial fibrillation [17] and cardiovascular disease (CVD) morbidity [18]. Increased adiponectin levels were also associated with an increase in risk for ischemic stroke [19]. In addition, no relationship between a high serum total adiponectin and CVD in type 2 diabetes was found [20]. Due to the complex balance between pro-and anti-inflammatory activity their pathophysiological and prognostic role in CVD still remains debated [21]. Currently, no data are reported about the association between serum levels of adiponectin and functional prognosis in patients with ischemic stroke. The purpose of this study was to assess the impact of serum adiponectin levels on functional prognosis in Chinese patients with ischemic stroke.

## Methods

### Patients

This was a prospective, observational cohort study. In this study, consecutive first-ever ischemic stroke patients without any pre-morbid handicap admitted to our hospital of Beijing, China, were identified. Patients were eligible for inclusion if they were with symptom onset within 48 h. The sample was determined by the research period, which was from December 2017 to December 2018. The study population was exclusively Chinese. Ischemic stroke was defined according to World Health Organization recommendations (defined stroke as a “neurological deficit of cerebrovascular cause that persists beyond 24 h or is interrupted by death within 24 h) [22] and were validated on the basis of magnetic resonance imaging (MRI), which had been performed within 24 h after admission. Exclusion criteria were: (1) malignant tumor and metabolic syndrome; (2) renal and/or liver insufficiency; (3) any surgical procedure within the previous 3 month; (4) inability to consent (e.g., dementia) and other neurological diseases (such as intracerebral hemorrhage, cerebral hemorrhage, Parkinson’s disease and Alzheimer’s disease) and (5) presence of cardiogenic shock, sepsis, pneumonia; acute coronary syndromes.

### Clinical variables and treatments

At admission, age, sex, body mass index (BMI) and vascular risk factors including: hypertension (high blood pressure noted in a patient’s medical history or patient under antihypertensive treatment), diabetes mellitus (glucose level > 7.8 mmol/l reported in the medical record or patient under insulin or oral hypoglycemic agents), hypercholesterolemia (total cholesterol level > 5.7 mmol/l reported in the medical history or patient treated with lipid-lowering therapy), atrial fibrillation (known or noted during the stay), smoking, and a history of transient ischemic attack (TIA) or stroke were recorded. BMI was calculated according to their height (m) and weight (kg): weight/height^2^. Comorbid conditions were determined from medication history and clinical assessment. Pre-stroke therapy, including oral anticoagulants, antiplatelet agents, antihypertensive treatment, and statins, as well as acute treatment (IV thrombolysis and/or mechanical thrombectomy) was also collected. The treatment of patients in the acute phase was determined by three doctors (Wang Z, Li B and Wang S) according to the factors such as the patient’s physical condition, onset time and clinical severity. The clinical stroke syndrome was determined applying the criteria of the Oxfordshire Community Stroke Project, while the stroke cause was determined according to the criteria of the TOAST (Trial of Org 10172 in Acute Stroke Treatment) classification [23]. The severity of stroke admission was assessed by the National Institutes of Health Stroke Scale (NIHSS) score (scores range from 0 to 42, with greater scores indicating increasing severity) [24].

### Neuroimaging

Diagnosis of stroke was based on the results of strict neurological examination (Brain computer tomography, magnetic resonance imaging (MRI), or both) according to the International Classification of Diseases, ninth revision. Thereafter, MRI was performed using a stroke protocol, including T1-, T2-, and diffusion-weighted imaging (DWI) sequences, and a magnetic resonance angiography. In those patients, DWI lesion volumes were determined by one experienced neurologist (Li B) unaware of the clinical and laboratory results. The infarct volume was calculated by using the formula 0.5 × a × b × c (where a is the maximal longitudinal diameter, b is the maximal transverse diameter perpendicular to a and c is the number of 10-mm slices containing infarct) [25].

### Follow-up and end points

All patients were received a 3-month follow-up. The primary end point of this study was good functional outcome of stroke patients, defined as an m-Rankin score (mRS) of 0 to 2 points. The poor functional outcome was defined as an mRS of 3 to 6 points [26]. Secondary end point was death from any cause. Outcome assessment was performed by two medical staff with a structured follow-up telephone interview with the patient or with the closest relative.

### Adiponectin assays

Serum concentration of adiponectin was routinely measured within the first 24 h after admission by a commercially available sandwich ELISA (Abcam Trading [Shanghai] Company Ltd. Shanghai, China). One hundred and twenty age, gender and BMI (± 0.1)-matched healthy volunteers from our hospital medical center were assigned to as the healthy control group. All control subjects were also clinically examined by a neurologist (not an author) to exclude the any sub-clinical stroke features. The median age of those control cases was 64 (IQR, 53–72) years and 56% were men.

### Statistical analyses

Results are expressed as percentages for categorical variables and as means (standard deviation, SD) and medians (interquartile ranges, IQRs) for the continuous variables, depending on the normal or non-normal distribution of data. Shapiro–Wilk tests were used for normal distribution test. Proportions and median values of baseline characteristics were compared between groups using the Chi-square and Mann–Whitney U test or two-tailed Student’s unpaired t-test when appropriate. Correlations between parameters were assessed by Spearman correlations.

Associations between adiponectin and either clinical severity at admission (dichotomized as NIHSS < 6 and NIHSS ≥ 6) [27], poor outcomes (defined by an mRS > 2) or mortality at 3-month after admission were analyzed using logistic regression to obtain odds ratios (OR) and 95% confidence intervals (CI). In multivariate analyses, significant factors which confirmed in the univariate analyses, including age, atrial fibrillation, IV thrombolysis and/or mechanical thrombectomy, NIHSS at admission, lesion volumes, stroke syndrome, serum levels of glucose, CRP and adiponectin were adjusted.

Second, the accuracy of adiponectin for predicting outcome of stroke was evaluated with receiver operating characteristic (ROC) curves and results were calculated with area under the curve (AUC) [28]. Thereby the area under the receiver operating characteristic curve (AUC) is a summary measure over criteria and cut-point choices. The adiponectin was dichotomized as high (≥ cut-off value) and low (< cut-off value). To test whether the adiponectin level improves score performance, we considered the two nested logistic regression models with NIHSS and adiponectin as compared with NIHSS only.

Lastly, cumulative overall survival rates were computed using the Kaplan–Meier method according to high and low adiponectin level and were compared using the log-rank test. Multivariate hazard ratios (HR) and 95% CI after adjusted for all significant predictors were assessed the Cox regression analysis. Statistical analysis was performed with SPSS for Windows, version 22.0 (SPSS Inc., Chicago, IL, USA) and the ROCR package (version 1.0–2). All testing was two tailed, and P values less than 0.05 were considered to indicate statistical significance.

### Ethics

Written informed consents were obtained from all patients; and, this study conformed to the principles of the Declaration of Helsinki was approved by the investigational review board of the First Affiliated Hospital of Xinjiang Medical University.

## Results

In this study, we recorded 232 stroke patients. The serum level of adiponectin was obtained in 227 patients (97.8%) with a median value of 7.0 μg/ml (IQR, 4.4–10.0 μg/ml), which was significantly higher (*P *< 0.001) than in those heathy controls (median: 4.9 [3.6–7.2] μg/ml). The median NIHSS scores on admission was 6 points (IQR 2 to 11). The patients received acute treatment were 11.9% for IV thrombolysis and 7.5% for mechanical thrombectomy. The characteristics of stroke patients are shown in Table 1. Furthermore, we found that serum adiponectin levels did not significantly differ (P = 0.38) between patients received antidiabetic drug therapy (N = 31) and patients did not receive antidiabetic drug therapy (N = 196).

**Table 1:** Characteristics of stroke patients according to stroke outcomes

	Total	Good outcomes	Poor outcomes	P^a^
N	227	169	58	–
Age, years	64 (53–72)	62 (51–70)	71 (62–83)	0.009
Sex-male	128 (56.4)	94 (55.6)	34 (58.6)	0.69
BMI, kg/m^2^	24.2 (22.8–26.4)	24.0 (22.7–26.3)	24.5 (23.2–26.8)	0.38
Prior vascular risk factors, n (%)				
Hypertension	145 (63.9)	102 (60.4)	43 (74.1)	0.059
Hypercholesterolemia	68 (30.0)	49 (29.0)	19 (32.8)	0.59
Coronary heart disease	57 (25.1)	42 (24.9)	15 (25.9)	0.88
Atrial fibrillation	49 (21.6)	31 (18.3)	18 (31.0)	0.043
Diabetes mellitus	53 (23.3)	38 (22.5)	15 (25.9)	0.60
Previous TIA	25 (11.0)	17 (10.1)	8 (13.8)	0.43
Smoking	55 (24.2)	40 (23.7)	15 (25.9)	0.74
Pre-stroke treatment, n (%)				
Antihypertensive treatment	130 (57.3)	95 (56.2)	35 (60.3)	0.58
Antidiabetic	31 (13.7)	23 (13.6)	8 (13.8)	0.97
Antiplatelet agents	72 (31.7)	52 (30.8)	20 (34.5)	0.60
Anticoagulants	23 (10.1)	18 (10.7)	5 (8.6)	0.66
Statins	51 (22.5)	37 (21.9)	14 (24.1)	0.72
Acute treatment, n (%)				
IV thrombolysis	27 (11.9)	26 (15.4)	1 (1.7)	0.011
Mechanical thrombectomy	17 (7.5)	16 (9.5)	1 (1.7)	0.053
Stroke etiology, n (%)				
Small-vessel occlusive	33 (14.5)	26 (15.4)	7 (12.1)	0.54
Large-vessel occlusive	41 (18.1)	31 (18.3)	10 (17.2)	0.85
Cardioembolic	75 (33.0)	56 (33.1)	19 (32.8)	0.96
Other	15 (6.6)	11 (6.5)	4 (6.9)	0.92
Unknown	63 (27.8)	45 (26.6)	18 (31.0)	0.52
Stroke severity, NIHSS at admission	6 (2–11)	5 (1–9)	9 (5–15)	< 0.001
DWI lesion, ml	15.2 (8.5–26.5)	13.2 (7.1–21.4)	20.9 (11.3–33.7)	< 0.001
Laboratory findings				
Glucose level, mmol/l	5.95 (5.53–6.63)	5.73 (5.35–6.33)	6.35 (5.94–7.15)	0.009
CRP, mg/l	5.6 (3.0–9.2)	4.9 (2.5–7.9)	7.0 (4.7–11.7)	< 0.001
Adiponectin, μg/ml	7.0 (4.4–10.0)	6.3 (4.0–8.9)	10.0 (7.0–13.9)	< 0.001
Stroke syndrome				
TACS	24 (10.6)	10 (5.9)	14 (24.1)	< 0.001
PACS	96 (42.3)	72 (42.6)	24 (41.4)	0.87
LACS	54 (23.8)	37 (21.9)	17 (29.3)	0.25
POCS	53 (23.3)	50 (29.6)	3 (5.2)	< 0.001

The results of categorical variable and continuous variable were presented as n (percentage) and median value (IQR). The mRS of 0–2 points was indicated as a good functional outcome, while 3–6 points was defined as poor outcome

*NIHSS* National Institutes of Health Stroke Scale, *TIA* transient ischemic attack, *IL-6* interleukin-6, *IQR* interquartile ranges, *mRS* modified Rankin Scale, *DWI* diffusion weighted imaging, *TACS* total anterior circulation syndrome, *PACS* partial anterior circulation syndrome, *LACS* lacunar syndrome, *POCS* posterior circulation syndrome, *CRP* C-reactive protein

^a^Chi-square and Mann–Whitney U test were applied for comparing the proportions and medians values between groups

### Serum levels of adiponectin and stroke severity

As a continuous variable, a positive correlation between NIHSS score and serum levels of adiponectin was reported (r[spearman] = 0.266; P < 0.001). As a categorical variable, 110 patients (48.5%) were defined as minor stroke (NIHSS < 6) and 117 (51.5%) were moderate-to-high clinical severity stroke. Serum levels of adiponectin in minor stroke were lower than that observed in patients with moderate-to-high stroke (6.0 [IQR, 3.8–9.1] μg/ml vs. 7.8 [5.3–11.2] μg/ml, P < 0.001). In multivariable models adjusted for age, sex, and other risk factors, adiponectin levels were associated with moderate-to-high stroke, and risk increased by 12% (OR = 1.12; 95% CI 1.03–1.25; P = 0.002). Furthermore, adiponectin was found to be associated with lesion size. A positive association between adiponectin and the infarct volume (r = 0.232, P < 0.001) had been found.

Based on the ROC curve, the optimal cutoff value of serum adiponectin levels as an indicator for diagnosis of moderate-to-high stroke was projected to be 7.0 μg/ml, which yielded a sensitivity of 63.6% and a specificity of 62.4%, with the area under the curve at 0.65 (95% CI 0.58–0.72).

### Serum adiponectin levels and functional outcome

**Fig. 1:**
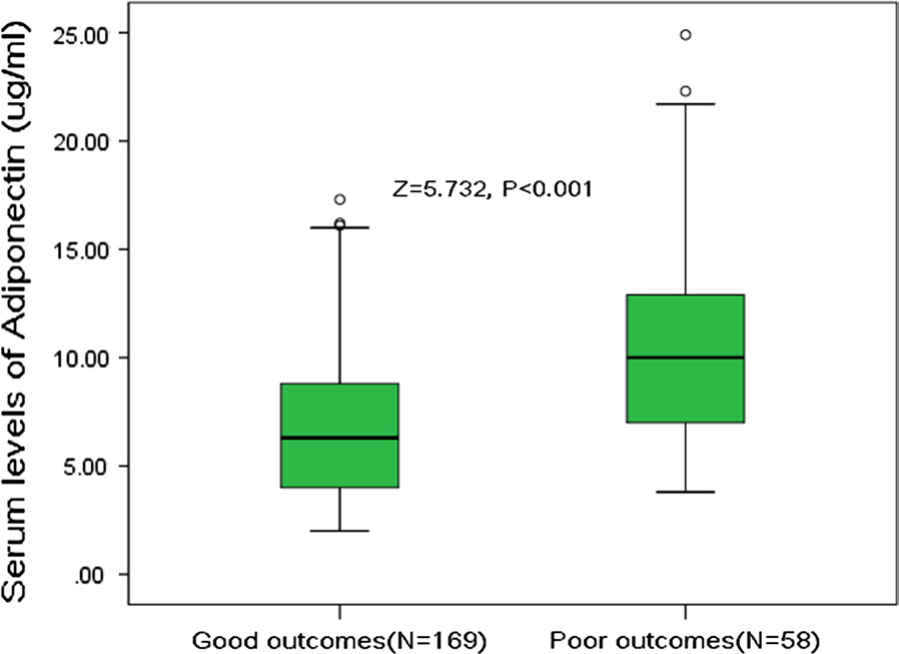
Serum levels of adiponectin in stroke patients with poor functional outcomes and good outcomes. A good outcome was defined as a mRS score of 0 to 2 points, while poor outcome was defined as 3–6 points. All data are medians and inter-quartile ranges (IQR). P values refer to Mann–Whitney U tests for differences between groups

At 3-month follow-up, 58 patients (25.6%) had poor functional outcomes whereas 169 patients (74.4%) had good outcomes. In the latter group, the median adiponectin level was lower than that observed in patients with poor outcomes (6.3 [4.0–8.9] μg/ml vs. 10.0 [7.0–13.0] μg/ml, P < 0.001) (Fig. 1). As shown in Table 1, stroke patients with poor outcome were more likely older, suffered from atrial fibrillation and have high blood levels of glucose and CRP but less likely to receive acute treatment. In addition, they also have a higher NIHSS and larger lesion size at admission.

In univariate and multivariate logistic regression analysis, for each 1 μg/ml increase of serum level of adiponectin the unadjusted and adjusted risk of poor functional outcomes would be increased by 36% (with the OR of 1.36 [95% CI 1.21–1.52], P < 0.001) and 24% (1.24 [1.13–1.37], P < 0.001; Table 2), respectively. Furthermore, as shown in Table 2, all risk factor (confirmed in the univariate analyses) included in multivariate analysis resulted to be predictors for poor functional outcomes, except for stroke syndrome and atrial fibrillation.

**Table 2:** Multivariate analyses of predictors of poor functional outcomes

	OR	95% CI	P
Age (increase per unit)	1.06	1.01–1.12	0.012
Atrial fibrillation (yes vs. no)	1.39	0.97–1.76	0.13
Stroke severity, NIHSS (increase per unit)	1.08	1.03–1.14	< 0.001
Lesion volumes (increase per unit)	1.02	1.00–1.04	0.021
IV thrombolysis and/or mechanical thrombectomy (yes vs. no)	0.64	0.51–0.87	0.025
Stroke syndrome (TACS vs other)	1.62	0.65–3.87	0.49
Glucose (increase per unit)	1.19	1.05–1.30	0.015
CRP (increase per unit)	1.45	1.11–1.76	0.003
Adiponectin (increase per unit)	1.24	1.13–1.37	< 0.001

Poor functional outcome was defined as an mRS > 2

*OR* odd ratio, *CI* confidence interval, *mRS* modified Rankin Scale, *NIHSS* National Institutes of Health Stroke Scale, *CRP* C-reactive protein, *TACS* total anterior circulation syndrome

Adjusted for significant factors which confirmed in Table 1, including age, atrial fibrillation, IV thrombolysis and/or mechanical thrombectomy, NIHSS at admission, lesion volumes, stroke syndrome, serum levels of glucose, CRP and adiponectin

**Fig. 2:**
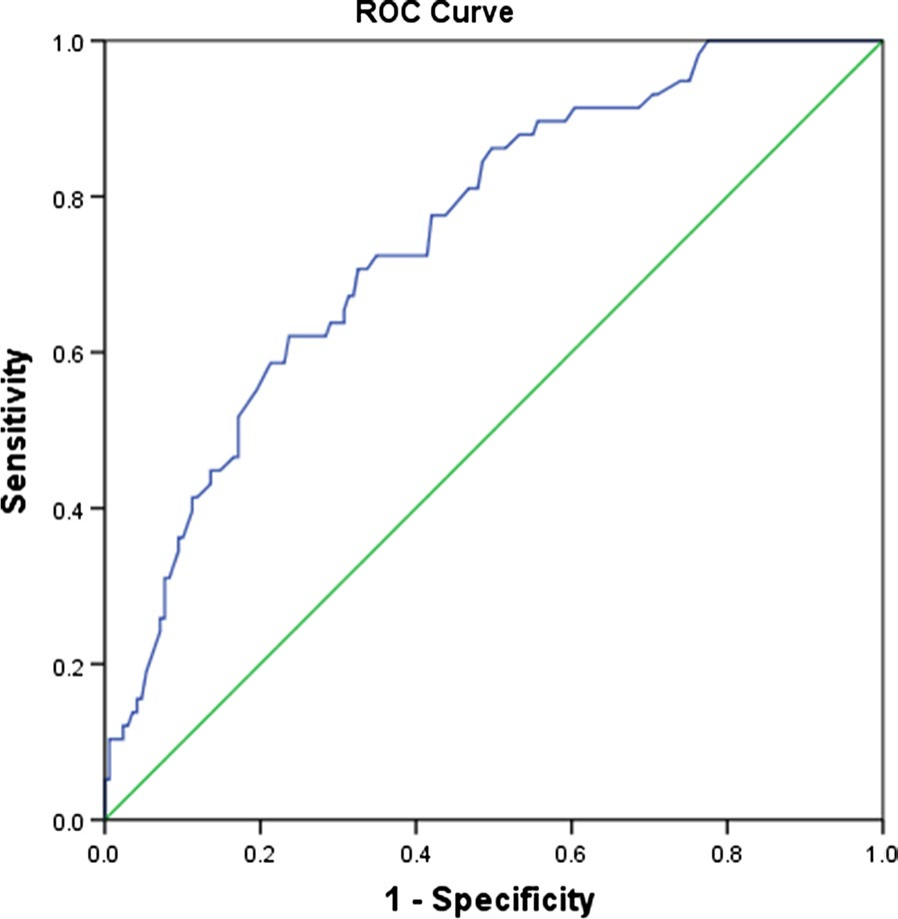
ROC curves were utilized to evaluate the accuracy of serum level of adiponectin to predict poor functional outcomes. Good outcome was defined as a mRS score of 0 to 2 points, while poor outcome was defined as 3–6 points

Based on the ROC curve (Fig. 2), the optimal cutoff value of serum adiponectin levels as an indicator for diagnosis of poor outcomes was projected to be 9.0 μg/ml, which yielded a sensitivity of 62.1% and a specificity of 72.3%, with the area under the curve at 0.75 (95% CI 0.68–0.82). With the AUC of 0.75, the adiponectin showed an improved discriminatory ability for poor outcome than age (0.63, 0.58–0.70; P < 0.001), CRP (0.68, 0.59–0.76; P = 0.002), and glucose (0.65; 0.57–0.73; P < 0.001), as well as in the range of NIHSS score (0.73, 0.65–0.80; P = 0.09). The combined model (adiponectin and NIHSS) improved the NIHSS score to predict poor outcomes (AUC of the combined model: 0.80; 95% CI 0.73–0.86; P < 0.001). Furthermore, classified according to cut-off value, the higher level of serum adiponectin (≥ 9.0 μg/ml) was a predictor of poor outcomes and the unadjusted and adjusted risk would be increased by 201% (with the OR of 3.01 [95% CI 1.94–4.21], P < 0.001) and 115% (2.15 [1.25–3.04], P < 0.001).

### Serum adiponectin levels and mortality

**Fig. 3:**
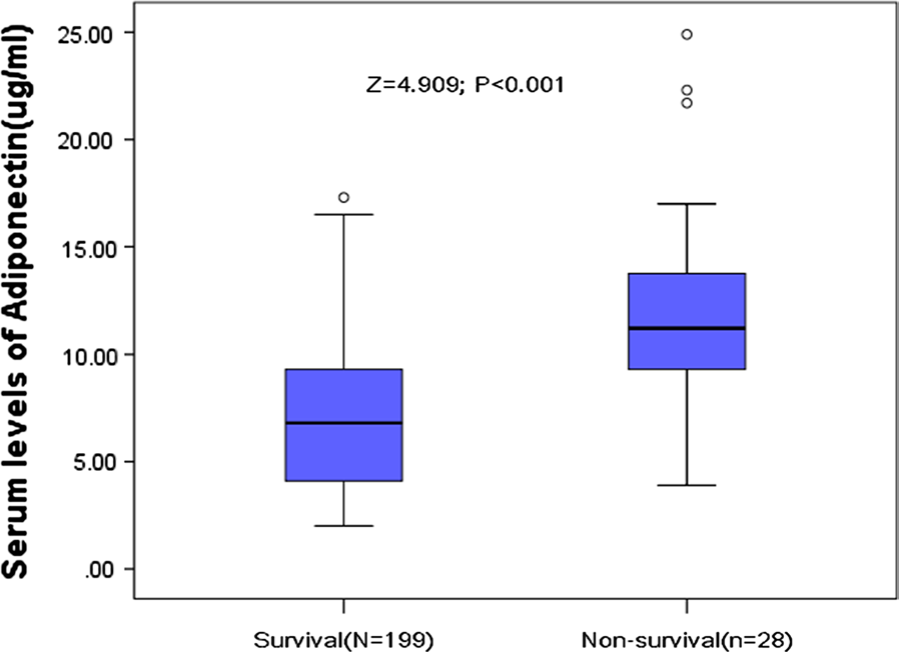
Serum levels of adiponectin in survivors and non-survivors of stroke. All data are medians and inter-quartile ranges (IQR). P values refer to Mann–Whitney U tests for differences between groups

Among the 227 patients, 28 of them (12.3%) died. Adiponectin levels in 28 patients who died were greater as compared with patients who survived (11.2 [IQR, 9.2–13.8] vs. 6.8 [IQR, 4.1–9.5] μg/ml; P < 0.001; see Fig. 3). In univariate and multivariate logistic regression analysis, for each 1 μg/ml increase of serum level of adiponectin the unadjusted and adjusted risk of mortality would be increased by 45% (with the OR of 1.45 [95% CI 1.30–1.62], P < 0.001) and 31% (1.31 [1.18–1.46], P < 0.001), respectively.

**Fig. 4:**
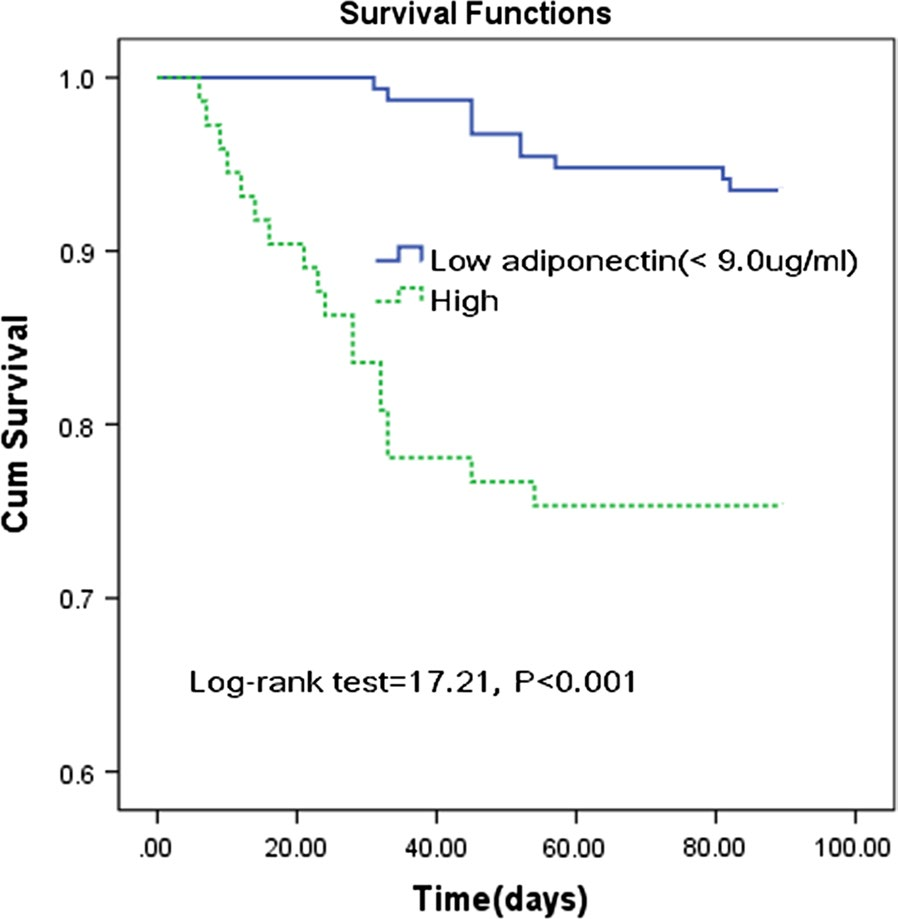
Kaplan–Meier analysis. The patients with high adiponectin serum levels had a higher risk of death compared to those with low serum adiponectin levels (log-rank test P < 0.001). High adiponectin serum level was defined as ≥ 9.0 μg/ml (cut-off)

Based on the ROC curve, the optimal cutoff value of serum adiponectin levels as an indicator for diagnosis of poor outcomes was projected to be 9.0 μg/ml, which yielded a sensitivity of 78.6% and a specificity of 72.9%, with the area under the curve at 0.79 (95% CI 0.70–0.88). The combination of adiponectin level and the NIHSS score had a higher discriminatory accuracy (AUC, 0.83; 95% CI 0.78–0.94) than the NIHSS score alone (0.78; 0.71–0.87; *P *= 0.006). Furthermore, classified according to cut-off value, the higher level of serum adiponectin (≥ 9.0 μg/ml) was a predictor of mortality and the unadjusted and adjusted risk would be increased by 276% (with the OR of 3.76 [95% CI 2.51–4.98], P < 0.001) and 156% (2.56 [1.42–3.99], P < 0.001). As shown in Fig. 4, the stroke patients were divided into two groups according to the cut-off value (high vs. low). Kaplan–Meier analysis suggested that the patients with high serum adiponectin levels had a higher risk of mortality than those patients with low level (log-rank test = 17.21; P < 0.001), and the risk of mortality would be increased by 221% (with the HR of 3.21 [95% CI 1.85–6.76], P < 0.001). Patients with high serum levels of adiponectin had a significantly shorter median survival time (24 vs. 49 days; P < 0.001) than those with low serum levels of adiponectin.

## Discussion

Inflammation is a hallmark of atherosclerosis, and the humoral markers (leptin and adiponectin) mediate the proinflammatory and anti-inflammatory responses, respectively, which may lead to ischemic stroke [29]. Here we assessed the association between adiponectin and functional prognosis in patients with ischemic stroke and the results showed that that high adiponectin levels were independently associated with both the clinical severity at admission and a poor functional prognosis in stroke patients.

Consistent with our findings, Nagasawa et al. [30] suggested that high levels of plasma total adiponectin can be a predictor of stroke mortality during the 17 months following an episode of acute stroke in patients, while another study confirmed that plasma total adiponectin values may help to classify stroke subtypes and predict neurological severity and functional outcome in ischemic stroke patients [31]. A previous study also showed that high plasma total adiponectin concentration was associated with mortality in patients with established atherosclerosis undergoing surgery for carotid artery stenosis, but not associated with ischemic events [32]. Furthermore, in patients with acute myocardial infarction but without previously known diabetes, high levels of adiponectin at discharge predicted total mortality [33]. Schrieks et al. [34] reported that adiponectin was prospectively associated with major adverse cardiovascular events and death in patients with type 2 diabetes and acute coronary syndrome, and an increase in adiponectin from baseline is directly related to death. In addition, Lekva et al. [35] suggested that high leptin/adiponectin ratio in pregnancy and in particularly in those with gestational diabetes mellitus were associated with an unfavorable CVD risk profile during follow-up.

Interestingly, Whitehall II study [36] showed that higher adiponectin levels were associated with a more favourable development of cardiovascular autonomic function in individuals with type 2 diabetes independently of multiple confounders, and another study showed that low plasma total adiponectin is related to an increased risk of 5-year mortality after first-ever ischemic stroke, independently of other adverse predictors [37]. In addition, one study reported that high total adiponectin was associated with a greater risk of incident disability and death, but not independently of these factors in the elderly [38]. Similarly, an observational cohort study suggested that blood total adiponectin levels are not related to further cardiovascular events in patients with type 2 diabetes [39]. In community-living elders, total and high-molecular-weight adiponectin showed U-shaped relationships with CVD [40]. Interestingly, Moreno et al. [41] found an unexpected deleterious role of adiponectin action/metabolism on atherosclerotic processes. Although adiponectin is a major adipocytokine that affects the pathogenesis of various cardiovascular diseases, its clinical significance in stroke remains controversial. More work should be carried out to assess the association between adipocytokine and cardiovascular diseases.

In this study, we found that serum adiponectin levels were higher in stroke than in those controls (P < 0.05). However, another study found that plasma adiponectin values did not significantly differ between the two groups (P = 0.836) [31]. In addition, the levels of adiponectin in atherosclerotic stroke patients were significantly lower compared with matched controls (P < 0.05) [42]. Compared with controls, ischemic patients display significantly reduced serum adiponectin levels [43]. During the acute phase of cerebral infarction, ischemic stroke patients, display significantly decreased adiponectin levels upon admission compared to control subjects, but these levels in ischemic stroke patients subsequently recover to basal levels [43, 44]. Furthermore, circulating levels of adiponectin associated with an increased risk of incident ischemic stroke were not supported by previous studies [45–47]. However, one study showed that higher plasma levels of total adiponectin were associated with an increased 10-year risk of ischemic stroke among healthy middle-aged men [48], while another study showed that increased serum total adiponectin was related to an elevated risk of ischemic stroke [49]. In this study, we could not confirm that the increased adiponectin concentrations within the first 24 h of a stroke represent a “response” of the human organism. Furthermore, adiponectin levels can be influenced by antidiabetic drugs [50, 51]. However, in this study, serum levels of adiponectin were not influenced by antidiabetic drugs.

To date, the pathophysiological mechanisms by which specific serum adiponectin play role in the stroke risk and prognosis are not fully understood. However, our study was an observational cohort study, and therefore, it was not possible to establish causality or potential treatment consequences. Previous studies had proposed some possible mechanisms. First, a reasonable possibility is that adiponectin resistance in metabolically active organs [52]. Second, natriuretic peptides may directly increase adiponectin expression [53] and natriuretic peptides were associated with stroke prognosis [23]. Masuch et al. [54] reported that elevated N-terminal pro-B-type natriuretic peptide (NT-proBNP) concentrations in mainly cardiac healthy individuals might relate to adiponectin signaling indicating even indirect cardio-protective effects for NT-proBNP. Third, under some chronic inflammatory conditions, adiponectin, rather than being an anti-inflammatory factor, exacerbates inflammation in several tissues and cell types [52, 55]. Adiponectin may be increased in proinflammatory conditions as a way to counteract systemic inflammation, potentially explaining its association with stroke prognosis [18]. Further efforts are needed to unravel the elusive role of adiponectin on stroke prognosis.

In this study, some shortcomings need acknowledge. First, we did not measure local adipose tissue of adipocytokines and/or serum expression of adipocytokines, such as retinol-binding protein-4, leptin, fatty acid-binding protein 4, omentin-1 and irisin. The association between serum adiponectin, other adipocytokines and stroke should be clarified. However, previous studies had showed the association between those adipocytokines and stroke [56–60]. Second, we only measured baseline serum levels of adiponectin, which might not represent the long-term levels of these markers. Nonetheless, plasma adipokine levels might remain stable over time [61]. The validity of the ELISA assay of adiponectin was also questioned by Bluher et al. [62] that reported significant differences between different commercially available assays. Third, we measured only total adiponectin levels, and not three isoforms. We also did not measure the activity of the adiponectin receptor and, thus, cannot directly test the adiponectin resistance hypothesis in the present study. Variant in the adiponectin gene was not tested and one study of Han population women from northern China, Chen et al. [63] demonstrated a relationship between the rs2241766 variant in the adiponectin gene and ischemic stroke risk. Fourth, we have to mention the small sample size of our population (N = 227), which affects the results of the multivariate analysis. In addition, our study cohort consists almost totally of Chinese. One study suggested that, in Japanese people, the westernization of their lifestyle might affect quantitative and qualitative changes in adiponectin and induce insulin resistance [64]. Thus, further studies in ethnically more diverse populations are warranted. Lastly, the cross-sectional study could not confirm any causal relationship. One study suggested that adiponectin level was not a causal factor of increasing stroke risk [65].

## Conclusions

To conclude, our results show that high adiponectin are associated with stroke severity and support the hypothesis that adiponectin can be serve as a biomarker of poor outcome after stroke. Future studies are needed to analyze more in depth and on larger populations the role of adiponectin and stroke prognosis. Whether adiponectin reduce treatment to normal levels would improve the stroke outcome need further investigate.

## Data Availability

Please contact corresponding author for data requests.

## Abbreviations

CVD: cardiovascular disease
MRI: magnetic resonance imaging
BMI: body mass index
TIA: transient ischemic attack
TOAST: Trial of Org 10172 in Acute Stroke Treatment
NIHSS: National Institutes of Health Stroke Scale
mRS: m-Rankin score
IQR: interquartile ranges
OR: odds ratios
HR: hazard ratios
CI: confidence intervals
ROC: receiver operating characteristic
AUC: area under the curve
NT-proBNP: N-terminal pro-B-type natriuretic peptide
